# Determination of conjugated protein on nanoparticles by an adaptation of the Coomassie blue dye method

**DOI:** 10.1016/j.mex.2019.09.015

**Published:** 2019-09-14

**Authors:** Mariana J. Oviedo, Katrin Quester, Gustavo A. Hirata, Rafael Vazquez-Duhalt

**Affiliations:** Centro de Nanociencias y Nanotecnología, Universidad Nacional Autónoma de México, Km 107 Carretera Ensenada-Tijuana, CP 22800, Ensenada, Baja California, Mexico

**Keywords:** Determination of protein coating on nanoparticles, Nanobiotechnology, Nanoparticle, Protein determination, Protein coating

## Abstract

A new accurate spectrophotometric method for protein determination on nanoparticles is described. The method is based on the Coomassie blue dye that binds to the basic and aromatic amino acid residues of proteins, especially arginine and lysine. A known amount of reagent dye was mixed with a variety of protein-loaded nanoparticles. Thereafter the unconjugated reagent was mixed with excess protein (bovine serum albumin) and titrated. In this method, the reacted dye on the protein coating of nanoparticle is directly determined, in opposite to the conventional method, in which the conjugated protein is determined as the difference between the non-conjugated protein found in the supernatant after centrifugation, and the total amount of protein originally used. This method is able to measure amounts of coated protein lower than 1 ppm.

• Simple and accurate method especially adapted for protein-coated nanoparticles.
• The amino acid residues of protein in the nanoparticle surface react with Coomassie brilliant blue dye.
• The unreacted dye is titrated with an excess of a standard protein.

## Graphical abstract

**Figure:**
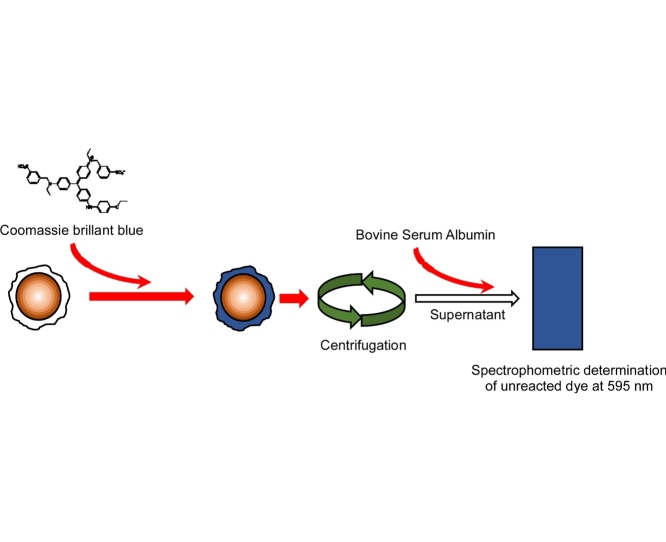

**Specification Table**

**Table:** 

Subject Area:	*Materials Science*
More specific subject area:	*Nanobiotechnology*
Method name:	*Determination of protein coating on nanoparticles*
Name and reference of original method:	*M.M. Bradford, A rapid and sensitive method for the quantitation of microgram quantities of protein utilizing the principle of protein-dye binding. Anal. Biochem. 72 (1976) 248*–*254.*
Resource availability:	https://www.bio-rad.com/webroot/web/pdf/lsr/literature/LIT33.pdf

## Background

Engineered nanomaterials, with their tunable physicochemical properties, are deeply transforming technologies in many areas including chemical manufacturing [1], food industry [2], medicine [3], personal care [4], electronics [5], sensors [6] and catalysis [7]. These nanomaterials are currently included in commercial products, such as cosmetics, sunscreens, sporting goods, automotive components and semiconductors, and their use is expected to grow significantly in the coming years.

The surface modification of nanomaterials such as nanoparticles should also provide new opportunities for their effective application in a wide variety of fields, especially peptide and protein modifications for nanomedicine [8,9]. The biological activity of proteins, such as recognition, catalysis or other biological effects, combined with the tunable properties of nanomaterials will, doubtless, revolutionize our lives. In the rapidly emerging field of Bionanotechnology, new materials that combine the diverse properties of biological molecules and nanostructured materials are being intensively investigated and developed.

Until now, the most common method to determine protein conjugated to the surface of nanoparticles is to measure remaining protein after bioconjugation, as performed for immobilized proteins. In brief, nanoparticles are mixed with a known amount of protein and, after reaction, nanoparticles coated with protein are collected by centrifugation, and the amount of residual protein in the supernatant is estimated using a conventional protein assay. The amount of conjugated protein is then calculated as the difference between the fed and the residual protein [[10], [11], [12]]. Nevertheless, this procedure cannot be used in nanoparticle preparations already and elsewhere synthetized.

Indirect and semi-quantitative methods have also been used to determine the protein coating on nanoparticles, such as: UV absorption spectroscopy [[13], [14], [15]], dynamic light scattering (DLS) [11,14], quartz crystal microbalance data (QCM) [14], transmission electron microscopy (TEM) [16], atomic force microscopy (AFM) [17,18], ultrafast X-ray scattering [19], Fourier transform infrared (FT-IR) spectroscopy [20], thermo-gravimetry [21] or enzymatic activity when enzymes are used [[21], [22], [23]]. In some cases, to verify the nanoparticle-protein interaction, another protein, such as green fluorescent protein (GFP) has been used [24]. The labeling of superficial protein with a flourophore has also been performed [25].

Attempts to directly measure attached protein by conventional determinations, as used for protein solutions, have been reported. Since protein content is measured using a UV–vis absorption method, a control for light absorbance by the suspended solid particles is needed, reducing the accuracy of the determination due to the not negligible source of interference [26].

The present method is a simple, rapid and accurate method to measure the protein content in coated nanoparticles.

## Method details

### Materials and protein coating

#### Nanoparticles (NPs) and carbon nanotubes (MWNTs)

Zinc oxide nanoparticles (110 nm particle size) 40% wt. in butyl acetate, *N*-Ethyl-*N*′-(3-dimethylaminopropyl)-carbodiimide (EDC), *N*-Hydroxysuccinimide (NHS) and (3-Aminopropyl)-trimethoxysilane (APTMS) were purchased from Sigma-Aldrich (St. Louis MO). Europium-doped hydroxyapatite powder was synthesized by a two-step process, consisting of the sol-gel method and subsequent annealing. The same method was used for the bismuth germanate (Bi_4_Ge_3_O_12_, BGO) and Y_2_O_3_:Eu^3+^ (with silica shell) nanoparticles [27]. A layered double hydroxide (Zn_0.75_Al_0.25_(OH)_2_(NO_3_)_0.25_·nH_2_O, LDH) was prepared by coprecipitation of zinc and aluminum salts [28]. Hybrid nanoparticles of zinc hydroxide chloride organo-modified with aspartate (ZHCl-Asp) and glycinate (ZHCl-Gly) were obtained by coprecipitation of zinc chloride salts with aspartic acid and glycine, respectively [28]. The carboxylated multi-walled carbon nanotubes (MWCNT−COOH) were prepared by chemical vapor deposition and purified by refluxing in an aqueous HNO_3_ solution (2.6 M) for 6 h, washed several times with distilled water and dried for 12 h at 80 °C [29]. A zeolite sample (Zn_1-.48_Cd_1-.60_ S_x_O_y_/H_8_(Al_8_Si_40_O_96_)·24H_2_O, MOR) was kindly donated by Dr. Vitalii Petranovskii.

Catalase, iso-1cytochrome c from *Saccharomyces cerevisiae* and bovine serum albumin (BSA) were obtained from Sigma-Aldrich (St. Louis MO). Laccase from *Coriolopsis gallica* was produced and purified as previously described [30], and versatile peroxidase was obtained from *Bjerkandera adusta* according Tinoco et al. [31].

#### Protein coating of nanoparticles and carbon nanotubes

A reaction mixture was prepared with either 50 mg of pure NP or 1 mg of carboxylated MWNT to which 50 μL of bovine serum albumin (8 mg mL^−1^) (BSA) and 10 μL of 0.1 M 1-ethyl-3-(3-dimethyl-aminopropyl) carbodiimide (EDC) were added. The mixtures were completed up to 1 mL with 10 mM Tris−HCl buffer (pH 5.5). The solutions were incubated at 37 °C for 5 h and then centrifuged at 8 000 rpm. The solutions of conjugated NPs and MWCNTs were washed two times with Tris−HCl buffer and three times with distilled water, and the supernatant was recovered by centrifugation.

#### Covalent coating of ZnO nanoparticles with different proteins

The method was also assayed with NPs covalently covered with different proteins. ZnO NPs (50 mg) were centrifuged at 14,000 rpm and washed first with acetone and then with toluene. The ZnO NPs were resuspended in 5 mL of toluene and sonicated for 10 min. The ZnO NPs were functionalized with (3-aminopropyl)-trimethoxysilane (APTMS) to introduce free amino groups on the nanoparticle surface. To the NPs suspension in toluene, 150 μL of APTMS and 100 μL of triethylamine were added drop by drop and kept shaking under nitrogen atmosphere for 12 h. Then, the suspension was centrifuged, washed three-times with ethanol and dried. The functionalized NP were resuspended in 1 mL of phosphate buffer (pH 6) and 100 μL of protein solution (10–30 mg/mL) were added (catalase, versatile peroxidase, laccase, cytochrome c or BSA). Then, 200 μL of 0.5 M EDC and 200 μL of 0.5 M NHS were added and kept for 1 h under soft shaking. The protein-covered NPs were then ultracentrifuged at 77 100*g* for 2 h and washed three times with a 100 mM phosphate buffer pH 6 to remove the unbonded protein. Then, the pellet was resuspended in 1 mL phosphate buffer.

#### Protein standard curve

The protein standard curve was obtained following the microassay indicated by the manufacturer (Bio-Rad, Protein Assay Dye Reagent). A set of dilutions ranging from 0 to 10 μg mL^−1^ of BSA protein was prepared. For each assay, 800 μL of BSA standard solution were mixed with 200 μL of Bio-Rad reagent and vortexed. Each dilution was then incubated at 25 °C for 5 min and absorbance read at 595 nm at room temperature using a spectrophotometer (Spectronic Genesyss 2, Spectronic Instruments). To obtain the standard curve, the absorbance measurements were plotted against protein concentration. This curve is consistent with these reported by the manufacturer.

(1) [protein] (μg mL^−1^) = 13.23 (Abs_595_)     r^2^ = 0.99

## Method description

Determination of conjugated protein. Fifty milligrams of coated or uncoated nanoparticles were suspended in 900 μL of distilled water and 100 μL of Bio-Rad reagent were added in a 1.5 mL Eppendorf tube. This amount of reagent dye is calculated to produce 0.5 absorbance units with an excess of protein. After 15 min incubation, all suspensions were centrifuged at 8000 rpm for 4 min. An aliquot of 500 μL of the supernatant of each suspension was transferred into a separate test tube to which an excess of BSA protein was then added (36 μL of BSA solution, 8 mg/mL), and the unconjugated reagent dye was spectrophotometrically determined at 595 nm. The reagent concentrations were transformed to BSA protein equivalents (μg mL^−1^) using the standard curve. Two control determinations were included: the first was prepared by mixing 50 mg of uncoated nanoparticles with 10 μL of EDC and 890 μL of distilled water; the second contained 50 mg of uncoated nanoparticles in 900 μL of distilled. To both controls were added 100 μL of Bio-Rad protein reagent. The amount of protein attached to the nanoparticles was estimated with the following equation.

(2) [protein in nanoparticles] (μg) = 13.23 [(Abs_595_ C) − (Abs_595_ NP)]

where "Abs_595_ NP" is the absorbance obtained from the assayed nanoparticles and "Abs_595_ C" is the absorbance of the blank without nanoparticles.

## Method validation

**Fig. 1:**
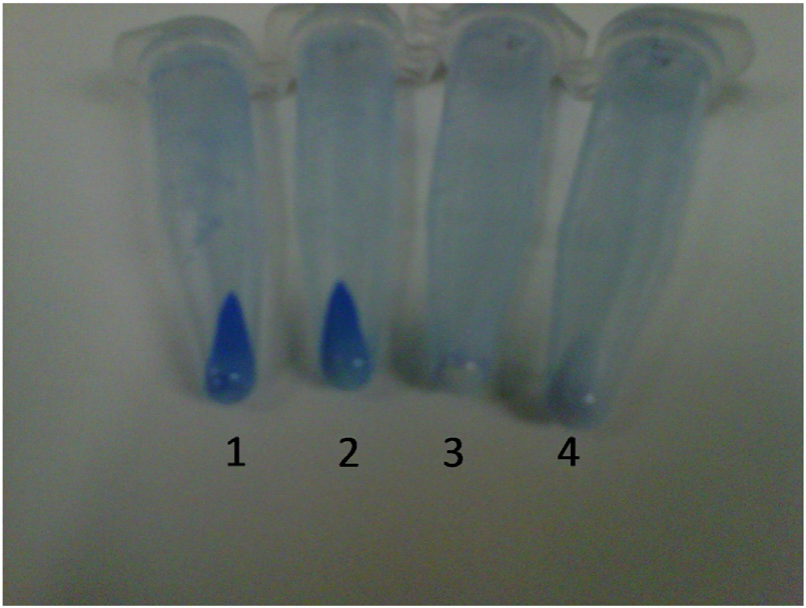
Different nanoparticle preparations after protein reagent treatment and later centrifugation. 1) HA:Eu^3+^ nanoparticles treated with BSA in the presence of EDC in 10 mM Tris HCL. 2) HA:Eu^3+^ nanoparticles treated with BSA in the absence of EDC in 10 mM Tris HCL. 3) HA:Eu^3+^ nanoparticles treated with EDC and without BSA in 10 mM Tris HCL. 4) HA:Eu^3+^ nanoparticles in 10 mM Tris HCL without BSA and EDC.

Eight nanoparticles, with different chemical nature, were coated with BSA: Europium-doped hydroxyapatite (HA:Eu^3+^), bismuth germanate (BGO), europium-doped yttrium oxide with silica shell (Y_2_O_3_:Eu^3+^), double layered hydroxide (LDH), hybrid nanoparticles of zinc hydroxide chloride organo-modified with aspartate (ZHCl-Asp) and glycinate (ZHCl-Gly), carboxyled multi-walled carbon nanotubes (MWCNT-COOH), and the mesoporous zeolite (MOR). To validate the method, a diversity of nanomaterials was selected including crystalline nanoparticles 20–45 nm, amino acid-modified materials, the widely used functionalized carbon nanotubes and the nanostructured natural material zeolite. All these materials have been previously characterized as cited in the materials section and shows quite different physicochemical properties. The amount of coating protein was determined, and the results are shown in Table 1. Control experiments with all nanoparticles showed low unspecific protein reagent adsorption (Fig. 1).

**Table 1:** Estimated values of conjugated protein in different materials.

Nanoparticle (NP)	Nanoparticle size (average, nm)	Protein load (μg protein/g NP)
HA:Eu^3+^	50	79.3 (±0.9)
BGO	6–12	134.9 (±62.3)
Y_2_O_3_:Eu^3+^ with SiO_2_	50–80	65.8 (±0.6)
MWCNT-COOH	Nd	126.1 (±10.3)
LDH	20	119.8 (±2.7)
ZHCl Gly	10	124.6 (±10.2)
ZHCl Asp	10	94.1 (±0.9)
MOR	8–12	116.5 (±9.2)

A modification of the standard method for protein determination in order to determine the amount of protein conjugated on the surface of nanoparticles was used. In brief, a known amount of reagent dye is added to the protein-coated nanoparticles. They are then centrifuged and the reagent remaining in the supernatant is titrated with an excess of protein. The protein coating on the surface of the nanoparticles is estimated by subtracting the unbound reagent dye from the amount initially added to the nanoparticles. The reagent dye concentration is transformed to BSA protein equivalents by the standard curve. Two controls are assayed, one with uncoated nanoparticles to estimate the possible unspecific adsorption of reagent dye, and another without nanoparticles to determine the initial amount of reagent dye. In the Bio-Rad Protein Assay, Coomassie blue binds to basic and aromatic amino acid residues, especially arginine [32], displaying a color change in response to protein concentration. This protein assay is derived from the Bradford method [33]. A color shift from 465 nm to 595 nm occurs in the presence of proteins. The absorbance increase at 595 nm follows the Lamber and Beer’s law, thus allowing accurate protein quantification.

It is important to point out that the Coomasie blue dye is without doubt the most widely used dye for protein determination. Since the first report by Bradford in 1976 [31] thousands of different proteins have been quantified using this reagent. The anionic blue form of the dye, which binds to protein, most strongly to arginine and lysine residues of proteins and also, to lesser extents, histidine and aromatic residues (tryptophan, tyrosine and phenylalanine). Nevertheless, in order to probe the method for different proteins. Zinc oxide NPs were covalently covered with five different proteins. Versatile peroxidase, catalase, laccase, cytochrome c and bovine serum albumin were covalently bond to the NPs and the protein content was determined (Table 2). The protein load varies from 58 to 150 μg of protein per g of ZnO NPs. The extent of conjugation depends on the number of free amino groups in the protein surface and on the reactivity of these groups at the reaction pH. These results clearly show that the method reported here could be used for any protein and any inorganic nanoparticle.

**Table 2:** Determination of protein content of ZnO nanoparticles covalently coated with different proteins.

Protein	Protein load (μg protein/g NP)
Bovine serum albumin (BSA)	146.8
Cytochrome c (Cyt C)	58.1
Catalase (Cat)	58.3
Laccase (Lac)	65.0
Versatile peroxidase (VP)	106.5

**Fig. 2:**
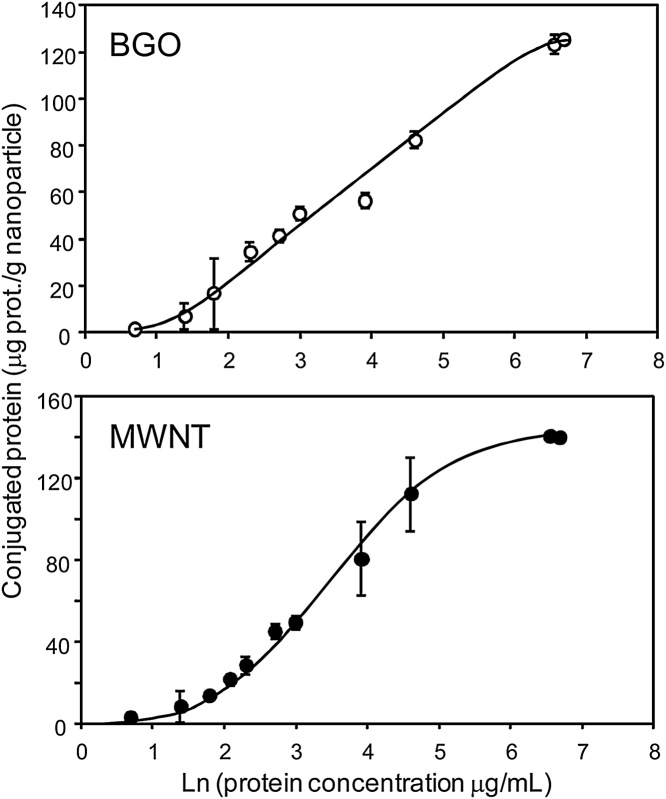
Extent of protein conjugation on the nanoparticle surface with different amounts of protein in the reaction mixture.

Saturation curves of protein coating were determined for BGO nanoparticles and MWCNT-COOH. The protein concentration in the reaction mixture was gradually increased and, as expected, the amount of protein attached to the nanoparticles and MWCNT also increase until saturation was reached. The saturation values were 125 μg of protein per g of BGO nanoparticles and 140 μg of protein per g of MWCNT−COOH (Fig. 2).

As in all analytical methods, this procedure may have interferences. The interferences for the protein reagent from Bio Rad are reported in the manufacturer's brochure. These interferences may be caused by the chemical-protein or the chemical-dye interactions. Contrarily to the manufacturer’s report, organic solvents, such as acetone, ethanol, methanol and acetonitrile, significantly affect this assay.

Coomassie assay is simpler, faster, and more sensitive than the Lowry and Biuret methods. In both, Lowry and Biuret methods, several reagent mixtures are needed making impossible the determination of protein bonded to the nanoparticles. To use these alternative methods is necessary to liberate first the protein from the nanoparticle conjugate. Moreover, when compared with these other methods, Coomassie dye is subject to less interference by common reagents and non-protein components of biological samples.

The bionanotechnology is a new field showing a very rapid progress with great scientific and technological opportunities. Bionanotechnology combines the unique properties of nanosized materials with the sophisticated activity of biological molecules. The development of new materials based on the biological molecules and nanostructured materials is rising for potential biomedical and industrial applications. The biological activity of proteins, such as recognition, catalysis or biological effects, combined with the tunable properties of nanomaterials will, doubtlessly, revolutionize our lives. Thus, it is imperious to have an easy and accurate method for protein determination on conjugated bionanomaterials.

This work presents a simple and accurate method to quantify the amount of protein conjugated to nanoparticles. This takes us a step further in the direction of being able to use protein-conjugated nanoparticles with more precision, due to an easier method of quantification of the protein attached to nanoparticles.

## References

[1] Zhao Q.Q., Boxman A., Chowdhry U. (2003). Nanotechnology in the chemical industry: opportunities and challenges. J. Nanopart. Res..

[2] Sozer N., Kokini J.L. (2009). Nanotechnology and its applications in the food sector. Trends Biotechnol..

[3] Boulaiz H., Alvarez P.J., Ramirez A., Marchal J.A., Prados J., Rodríguez-Serrano F., Perán M., Melguizo C., Aranega A. (2011). Nanomedicine: application areas and development prospects. Int. J. Mol. Sci..

[4] Morganti P. (2010). Use and potential of nanotechnology in cosmetic dermatology. Clin. Cosmet. Invest. Dermatol..

[5] Haselman M., Hauck S. (2010). The future of integrated circuits: a survey of nanoelectronics. Proc. IEEE.

[6] Yeom S.-H., Kang B.-H., Kim K.-J., Kang S.-W. (2011). Nanostructures in biosensor: a review. Front. Biosci..

[7] Bell A.T. (2003). The impact of nanoscience on heterogeneous catalysis. Science.

[8] Knopp D., Tang D., Niessner R. (2009). Review: bioanalytical applications of biomolecule-functionalized nanometer-sized doped silica particles. Anal. Chim. Acta.

[9] De La Rica R., Matsui H. (2010). Applications of peptide and protein-based materials in bionanotechnology. Chem. Soc. Rev..

[10] Voltan R., Castaldello A., Brocca-Cofano E., Altavilla G., Caputo A., Laus M., Sparnacci K., Ensoli B., Spaccasassi S., Ballestri M., Tondelli L. (2007). Preparation and characterization of innovative protein-coated poly(methylmethacrylate) core-shell nanoparticles for vaccine purposes. Pharm. Res..

[11] Zhou W., Schwartz D.T., Baneyx F. (2010). Single-pot biofabrication of zinc sulfide immuno-quantum dots. J. Am. Chem. Soc..

[12] Cui Y., Chen X., Li Y., Liu X., Lei L., Xuan S. (2012). Novel magnetic microspheres of P (GMA-b-HEMA): preparation, lipase immobilization and enzymatic activity in two phases. Appl. Microbiol. Biotechnol..

[13] Vinayaka A.C., Thakur M.S. (2011). Photoabsorption and resonance energy transfer phenomenon in CdTe-protein bioconjugates: an insight into QD-biomolecular interactions. Bioconjug. Chem..

[14] Choi S.Y., Jeong S., Jang S.H., Park J., Park J.H., Ock K.S., Lee S.Y., Joo S.W. (2012). In vitro toxicity of serum protein-adsorbed citrate-reduced gold nanoparticles in human lung adenocarcinoma cells. Toxicol. In Vitro.

[15] Giri J., Diallo M.S., Simpson A.J., Liu Y., Goddard W.A., Kumar R., Woods G.C. (2011). Interactions of poly(amidoamine) dendrimers with human serum albumin: binding constants and mechanisms. ACS Nano.

[16] Yang Y., Jia Y., Gao L., Fei J., Dai L., Zhao J., Li J. (2011). Fabrication of autofluorescent protein coated mesoporous silica nanoparticles for biological application. Chem. Commun..

[17] Valo H., Kovalainen M., Laaksonen P., Häkkinen M., Auriola S., Peltonen L., Linder M., Järvinen K., Hirvonen J., Laaksonen T. (2011). Immobilization of protein-coated drug nanoparticles in nanofibrillar cellulose matrices. Enhanced stability and release. J. Control. Release.

[18] Ge C., Du J., Zhao L., Wang L., Liu Y., Li D., Yang Y., Zhou R., Zhao Y., Chai Z., Chen C. (2011). Binding of blood proteins to carbon nanotubes reduces cytotoxicity. Proc. Natl. Acad. Sci. U. S. A..

[19] Ibrahimkutty S., Kim J., Cammarata M., Ewald F., Choi J., Ihee H., Plech A. (2011). Ultrafast structural dynamics of the photocleavage of protein hybrid nanoparticles. ACS Nano.

[20] Samanta B., Yan H., Fischer N.O., Shi J., Jerry D.J., Rotello V.M. (2008). Protein-passivated Fe(3)O(4) nanoparticles: low toxicity and rapid heating for thermal therapy. J. Mater. Chem..

[21] Hommes G., Gasser C.A., Howald C.B.C., Goers R., Schlosser D., Shahgaldian P., Corvini P.F.X. (2012). Production of a robust nanobiocatalyst for municipal wastewater treatment. Bioresour. Technol..

[22] Qhobosheane M., Santra S., Zhang P., Tan W. (2001). Biochemically functionalized silica nanoparticles. Analyst.

[23] Ha T.H., Jeong J.Y., Chung B.H. (2005). Immobilization of hexa-arginine tagged esterase onto carboxylated gold nanoparticles. Chem. Commun..

[24] Valo H.K., Laaksonen P.H., Peltonen L.J., Linder M.B., Hirvonen J.T., Laaksonen T.J. (2010). Multifunctional hydrophobin: toward functional coatings for drug nanoparticles. ACS Nano.

[25] Chithrani B.D., Chan W.C. (2007). Elucidating the mechanism of cellular uptake and removal of protein-coated gold nanoparticles of different sizes and shapes. Nano Lett..

[26] Stayton I., Winiarz J., Shannon K., Ma Y. (2009). Study of uptake and loss of silica nanoparticles in living human lung epithelial cells at single cell level. Anal. Bioanal. Chem..

[27] Taxak V.B., Khatkar S.P., Han S.D., Kumar R., Kumar M. (2009). Tartaric acid-assisted sol-gel synthesis of Y2O3:Eu3+ nanoparticles. J. Alloys Compd..

[28] Carbajal Arizaga G.C. (2012). Intercalation studies of zinc hydroxide chloride: ammonia and amino acids. J. Solid State Chem..

[29] Lin Y., Rao A.M., Sadanadan B., Kenik A.E., Sun Y.P. (2002). Functionalizing multiple-walled carbon nanotubes with aminopolymers. J. Phys. Chem. B.

[30] Tinoco R., Pickard M.A., Vazquez-Duhalt R. (2001). Kinetic differences of purified laccases from six Pleurotus ostreatus strains. Lett. Appl. Microbiol..

[31] Tinoco R., Verdin J., Vazquez-Duhalt R. (2007). Role of oxidizing mediators and tryptophan 172 in the decoloration of industrial dyes by the versatile peroxidase from Bjerkandera adusta. J. Mol. Catal. B Enzym..

[32] Compton S.J., Jones C.G. (1985). Mechanism of dye response and interference in the Bradford protein assay. Anal. Biochem..

[33] Bradford M.M. (1976). A rapid and sensitive method for the quantitation of microgram quantities of protein utilizing the principle of protein-dye binding. Anal. Biochem..

